# Effects of the COVID-19 Pandemic on the Success of Traditional Small and Medium Enterprises (SMEs): An Investigation of the Footprints of Economic Crisis Attributable to COVID-19

**DOI:** 10.3389/fpsyg.2022.924340

**Published:** 2022-10-13

**Authors:** Anum Khaliq, Shunaid Ali, Ziyi Chen, Sehrish Kanwal, Farina Khan, Abdul Aziz Khan Niazi, Leping Chen

**Affiliations:** ^1^Department of Economics, Shah Abdul Latif University, Khairpur, Pakistan; ^2^School of Finance and Trade, Wenzhou Business College, Wenzhou, China; ^3^University of Central Punjab, Lahore, Pakistan; ^4^School of Public Administration, Nanjing Agriculture University, Nanjing, China; ^5^Institute of Business and Management, University of Engineering and Technology, Lahore, Pakistan

**Keywords:** success in e-business, customer satisfaction, absorptive capacity, customer knowledge management, job satisfaction, COVID-19

## Abstract

The COVID-19 pandemic created a significant economic decline and altered market behavior, forcing buyers and dealers online. The traditional local market merchants are not fully equipped with e-commerce business techniques and strategies, which is a barrier to their e-commerce behavior and success. The study aims to help small-medium firms adapt to an uncertain economic environment instead of reducing or shutting down business-like in Pakistan. From health to education, economy to domestic and social protection, various researches have been done since 2020. The researcher used primary data sources and did a Quantitative study after collecting the 240 samples size of data from the successful e-commerce players of Pakistan. The results confirm that customer satisfaction is essential for entrepreneurs to succeed, as customers were not satisfied with online shopping during COVID-19. Customer knowledge management (CKM) and job satisfaction (JS) are studied as potential and realized capacity variables. CKM act as a strategic asset to collect and assimilate the external customer knowledge. In contrast, satisfied employees act as a valuable asset that dynamically responds to changing customer needs and business environment by efficiently utilizing their knowledge and skills and reaching business success which is mirrored in customer satisfaction. Conclusive results enable practitioners to perceive the business success during economic crises in the organization’s absorptive capacity.

## Introduction

COVID-19 is a pandemic that outbreaks in Wuhan, China, in 2019 but still yet affects the emotional, social, personal, and professional lives of the people worldwide. These sound effects have disrupted consumer behavior, business models, and supply chain in trades such as tourism, manufacturing, consumer retail, and transportation. Policies taken by the worldwide governments to control the global health crises of coronavirus either triggered and sustained the global recession caused by the 2020 pandemic (Ozili and Arun, 2020). Companies need to innovate in their strategies, structure, culture, and management to stay in the game as it supports them in avoiding uncertainty and calamities better. High uncertainty and deep socioeconomic crises, like that initiated by a coronavirus, can negatively affect the entrepreneurial behavioral intention (Ruiz-Rosa et al., 2020), but organizations with absorptive capacity can devise the crisis management strategic decisions and processes to set an organization toward recovery from recession instead of shutting down.

By 2020, the world is facing global social, human, and economic crises caused by the coronavirus, attacking societies at their core and shrinking the global economy by up to –3% in 2020. Along with the primary public health threat, it is increasingly becoming the biggest global financial and economic threat. As a result of pandemic crises, the accumulative loss to global GDP over 2020 and 2021 could be around $ 9 trillion which is greater than the combined economies of Japan and Germany (Gita Gopinath, Chief Economist of the IMF, 2020 reported in 2020), informed that if the virus continued to grow in 2021, the global GDP might fall by an additional 8% compared to the baseline scenario of the IMF. COVID-19 recession is characterized by the greatest uncertainty initiated by a supply and demand shock triggered due to lockdown, interruption in trade flow, and high unemployment, which significantly decline personal income (Balcilar, 2020). In accord with Dai et al. (2021), due to the outbreak of COVID-19 government-imposed quarantine restrictions cast a substantial toll on small and medium enterprises (SMEs), which act as a backbone of global economies (Karadag, 2015). For instance, in high-income countries, it contributes to above 55% of GDP and above 65% of the total employment; in middle-income countries, it contributes to over 95% of employment and about 70% of GDP, while in low-income countries, it accounts for over 70% of the total employment and over 60% of GDP (Zafar and Mustafa, 2017). Similar to many other countries, SMEs set up nearly 90% of all the businesses in Pakistan, approximately 40% share in the annual GDP, and employ 80% of the non-agricultural labor force (SMEDA Pakistan; Shah, 2018). COVID-19 has suffered Pakistan’s economy, severely affecting the small and medium businesses (Shafi et al., 2020). In addition, firms are facing a 38.04% reduction in sales and 41.85% reduction in profit; to handle the situation, they are implementing the tactics of completely and partially shutting down their business activities (31 and 19%, respectively), reducing the salaries of the staff (12%), applying for loans (18%), and laying off employees (43%).

Moreover, more than 83% of Pakistani small-medium enterprises were neither equipped nor had any proposal to drive such a critical situation. Indeed, an online survey conducted by SMEDA (2020) reported that 95% of businesses had practiced a decline in operation, 48% of enterprises have laid-off workers, and 89% of businesses are facing financial distress, while only 26% of enterprises plan to callback the laid-off staffs in a month. It is coherent that all the above-mentioned circumstances enhance the economic downfall as it increases unemployment and depletion in income. We argue that instead of firing employees, taking loans at high-interest rates, and limiting business activities, traditional small-medium enterprises (TSMEs) need to evolve their business strategies with the environmental changes caused by the pandemic. The environmental crises of COVID-19 have turned the entire world upside down, but it has shifted the TSMEs toward web entrepreneurship and lifted it. The world is in lockdown, which enables the entrepreneurs and customers toward online business and shopping. It develops a competitive market on the web because of quarantine restrictions entrepreneurs’ have no other choice but to market and sell their products online.

Furthermore, isolation regulations change consumers’ behavior and permit them to “go digital.” In this regard, United Nations Conference on Trade and Development reported in March 2021 that in the year 2020, COVID-19 had led to a 3% surge in global e-commerce trade. While in Pakistan, the size of the web business in the first quarter of 2021 has increased to Rs. 96 billion compared to Rs. 71 billion in quarter 1 of 2020 (NECC, 2021).

Due to the epidemic, small and medium-sized traditional businesses are now forced to go online, forcing them to compete with the largest online businesses in Pakistan, owned by Ali Baba, such as Jamshaid and Darazpk, which previously advertised on Facebook and Instagram, but now the pandemic enforces the small and medium traditional entrepreneurs to go online. For that, traditional Pakistani retailers are trying to enter a new market to perform e-commerce activities by using applications such as Tiktok, Whatsapp, and Youtube for advertising their products and services. But Afridi et al. (2021) stated that they failed to satisfy the customers. During online shopping, 47.50% of Pakistani customers face low-quality products, and 26.40% of customers face high price issues. This indicates that TSMEs need to understand the ways and strategies of successful e-commerce business as e-commerce expands the customer range and market. COVID-19 has created an opportunity for entrepreneurs to create the demand for their products and services by satisfying the developing and existing online customers. It is a highly challenging and competitive environment for TSMEs as lockdown mitigated the economic activity. Hence, an entrepreneur who fully acknowledges their customers and their needs and puts the efforts into evolving their systems, strategies, and employees to better cope with the changing market environment can gain success in e-commerce during COVID-19, which changes the behavior of the consumers. Environmental changes initiated the forceful emergence of e-commerce which challenges TSMEs of Pakistan to understand the new market to satisfy the customers to become the forgoing business player and reach approximately pre-pandemic crisis stability.

In organizations, it plays a key role in evolving and responding to environmental changes to progress the business success. Cohen and Levinthal (1990) commercialized the theory of absorptive capacity that it is a firm’s intrinsic capacity to observe, realize and exploit the value of external knowledge. Zahra and George (2002) propose that an organization’s dynamic capability concerning the knowledge formation and consumption to gain and sustain business success has subsets, i.e., potential and realized capacity. Customer knowledge is a strategic asset and source of gaining success in the competitive market, and it is efficiently utilized through customer knowledge management (CKM). It is used to determine an organization’s progress in the context of customer satisfaction. In firms, employee job satisfaction is reflected as a firm’s dynamic factor in efficiently responding to the varying customer needs in a changing business environment.

The major goal of this study is to see how absorptive capability relates to TSMEs’ e-commerce success during a recession. Second, this research demonstrates that customer knowledge management and job satisfaction are critical indicators of a TSME’s potential and realised absorptive capacity, and that customer satisfaction, rather than limiting or shutting down business activity, stimulates e-commerce success during the current economic crisis

## Literature Review

Business models depict an organization’s current or future condition, illustrating some aspects of how they conduct business. COVID-19 has evolved into a pandemic, beginning as tiny clusters of transmission that merged into bigger clusters in several nations, eventually leading to global transmission (Bodrud-Doza et al., 2020). In a couple of days, the entire scenario had profoundly affected the lives of individuals all around the world. The public was confronted with a new, uncertain, and fast changing environment (Rodríguez-Rey et al., 2020). As long as they are carefully integrated into the innovation process, new and improved business models can mediate between those elements and the increased performance of businesses (Di Vaio et al., 2020). Organizations have little control over the appearance or spread of the global COVID-19 pandemic; however, they must act properly and rethink (or rebuild) their business structures. Organizational mechanisms for knowledge exchange at corporate governance and specialized technological means play an important role in boosting firms’ performance. The process of discovering how new technologies might improve business models can begin within an organization.

In Pakistan, E-commerce is not a new concept as it started in 2001. Still, with the progression in information technology, it is exploring and fast-growing new business areas such as online banking, financial instrument trading, and freelancing services. Certainly, COVID-19 has changed the behavior of the Pakistani market and forcefully compelled them toward online. It shows a glance that the e-commerce contribution of the Pakistani market to the global growth rate was 26% in the year 2020, though in the first quarter of 2021, the growth rate is reported to be over 35%. COVID-19 necessitates the entrepreneurs to plan the strategies to efficiently manage and minimize the adverse effects of the pandemic to build back better economic and social life. Thus, entrepreneurs started to find ways and go digital to minimize the economic downfall. As a result, businesses confront several problems, such as ensuring customer satisfaction and gaining a grasp of the new market and clients. Client pleasure is the key aim of a business, even since COVID-19 has altered customer behavior from physical to digital (Priyono et al., 2020). In Pakistan, e-commerce is relatively a challenging phenomenon from both perspectives of producers and consumers, i.e., difficult for producers to online satisfy the customers to gain their trust and loyalty. In contrast, customers are confused with trusting the producer and waste money by spending on poor-quality products.

To satisfy the customers with desirable goods and services, organizations need to manage their knowledge about customers. According to Attafar et al. (2013), organizations who want to produce the customer’s expected products take a more comprehensive management approach, i.e., CKM. Customer relationship management and knowledge management are combined in CKM. Data was collected *via* random sampling, and the findings proved that CKM is a source of generating customer-oriented creative goods, which has a beneficial impact on consumer happiness and loyalty to the brand. In accord with Khasawneh and Alazzam (2015), CKM strategy emphasizes getting maximum advantage from customer knowledge; and providing customers with continuous improved products and services to maximize their satisfaction level. Mehmood and Zain ul Abedin (2017) investigated the influence of CKM on Customer Satisfaction (CS); the authors did a quantitative study and selected the hotel industry of Gujranwala, Pakistan, for the study. Their results showed that CKM enhances the capacity of an organization to serve the customers with their expected products and services and qualifies the company to foster decision-making on reliable information, which keeps the customers satisfied with the organization. Yaghoubi et al. (2011) enlighten that CKM supports the organizations to keep the existing customers and manage the new ones. Iranian researchers have studied the influence of CKM on customer satisfaction, customer value, and customer interaction in the automotive industry. Their research outcomes revealed that CKM could good evaluate customer satisfaction which predicts the firm’s market share and profitability. Adrutdin et al. (2018) explored the role of CKM in gaining student satisfaction by targeting the Malaysian Institute of Industrial Technology. According to their results, CKM eases an organization to understand the extent to which customer perceive and analyze the features of the product and services; and facilitate them with new direction and strategy to head customer satisfaction which is reflected in their loyalty toward the organization. Based on the above literature following hypothesis is generated to test:

***H1:*** *E-business customers are more satisfied during COVID-19 crises if they have more access to customer knowledge management systems*.

Customer satisfaction is highly affected by the job satisfaction of the employees. Daniel et al. (2012) defines the term satisfaction in business as an individual receiving more than or equal to their desires and expectations, eventually perceiving happiness and motivation. That condition exhibits their satisfaction whether customer or employee. Furthermore, the researcher stated that business accomplishments are the subsequent outcome of employee job satisfaction, but it is mirrored in customer satisfaction. Leah (2005) empirically studied the relation of employee job satisfaction to customer satisfaction in the call center industry that assists their veterinary clients; their study reveals that satisfied employees can better deliver services to the customers by exploiting their skills and learning and serving them efficiently to ensure their satisfaction. In Swamy et al. (2015), employees satisfied with their jobs are the key assets of an organization that the organizations use to achieve their goal, i.e., customer satisfaction. Researchers offer a new model.

Quality of work-life is the extent to observe the employee satisfaction with the job and collected the data from the employees of Mechanical manufacturing small-medium enterprises. In this regard, Al Kurdi et al. (2020) conducted a study and collected primary data from the employees who functioned for service organizations in Jordon, while secondary data was composed of interconnected preceding studies. Employees who are happy with their jobs are more likely to put in the necessary effort and training to supply personalized products and services over time and retain important clients (Didier et al., 2021). According to Amoopour et al. (2014), pleased employees are more dedicated to their employers and provide benefits to their businesses. Furthermore, satisfied employees play a crucial role in satisfying customers to gain success in the business, and it is measured through reward and employee communication. Khalaf et al. (2013) inspected how customer satisfaction is obtained from the perception of employee satisfaction. They found that if the employee is not satisfied with the job, whether working in a small or a large scale business, the company cannot gain customer satisfaction, affecting its productivity.

Moreover, in intense market competition, organizations need to understand the importance of employee satisfaction. The organization gains employee loyalty and encourages them to understand consumer wants and expectations in order to build goods and services that meet those needs and expectations (Branicki et al., 2018). Jeon and Choi (2012), the relationship between customer satisfaction and employee satisfaction is significant but not mutual. Findings reveal that customer satisfaction depends on the behavior of serving employees with them in terms of cooperation and understanding. Employee satisfaction is related to salary, supervisors’ behavior, benefits, and work environment. The general objective of their research was to observe whether the association between employee satisfaction and customer satisfaction is unilateral or bilateral based on dyadic data.

In this regard, Chinwe Monica and Abdul-Rasaq (2017) stressed that no organization could survive without the support of human resources that take part in achieving the organization’s set targets; researchers empirically studied the hotel industry of Nigeria and reported that the more satisfied an employee in terms of salary, supportive working environment and conditions, reward system, job security the more efficiently an employee perform their job and produce quality products and services to ensure company’s profitability and success through satisfying customers. Singh et al. (2020) looked at employee satisfaction on customer satisfaction in the hotel industry. According to their findings, contented, happy, and energized employees perform effectively at work by competently applying their learning and understanding of consumers, as well as bringing customer pleasure and productivity to the company (Cerami et al., 2020). Customer-centric organizations believe that customer loyalty depends on employees’ happiness (Sankar, 2019). The researcher measured customer satisfaction through customer retention intention, while employee satisfaction is measured through working conditions and management style in the setting of the car showroom industry. Their results reveal a positive and direct connection between employee satisfaction and customer satisfaction, expressed in terms of rewards and loyalty, respectively; further trained employees with satisfaction can better employ their skills and understanding to provide quality products and services. Singh et al. (2017) researched to explore the influence of employee job satisfaction on customer satisfaction. Researchers selected top and middle managers from the five hotels in Kuala Lumpur, Malaysia, as respondents. Their conclusive results demonstrate that efficient and effective employed operations can convey higher quality products and services that can excel customer satisfaction and increase a firm’s effectiveness. Ahmad and Raja (2021) investigated 440 employees of an Indian commercial bank to understand the significance of employee job satisfaction-customer satisfaction. Their study supported that a satisfied employee is energetic and motivated to perform their job more powerfully and head an organization to success by satisfying customers. Based on the overhead literature, we can develop the following statistical hypothesis:

***H2:*** *Customer satisfaction is more likely to be achieved amid the COVID-19 crisis if employees have a high degree of job satisfaction*.

### Customer Satisfaction and E-Commerce Success

COVID-19 has transferred the market competition from the traditional to the online domain. In contrast, technological advancements acknowledged the customers’ variety of products and services in terms of quality and prices and significantly affected their perceptions, needs, and expectations. Only those organizations will progress and prosper who think up techniques and processes to meet fluctuating customer needs, and online satisfy them moderately better than their rivals. When the company achieves positive outcomes that let it be profitable and competitive among rivals and in the market, it is believed that it is achieving success and accomplishments; hence, those economic outcomes are revenues, growth in sales, market share, cash flows, company survival, general performance and growth of the company (Murphy et al., 1996; Crowell, 1998; Richard et al., 2009; Achtenhagen et al., 2010; Wach, 2010; Makhbul and Hasun, 2011; Fried and Tauer, 2015; Zhou et al., 2017). According to Rahimić and Uštović (2012), entrepreneurs need to satisfy their customers to gain and preserve long-term success in business. Their research objective was to identify that customer satisfaction is a key factor differentiating companies from competitors. Data were collected from the 44 companies operating in the region of Herzegovina and Bosnia, and the researchers presented the application of the Customer-Oriented sales model. Their research findings illustrate that companies need to keep customer satisfaction as a primary motive to certify their long-term survival and success in the era of globalization. Later, customer satisfaction enhances their sales and profit ratio and brings new customers. Additionally, if the companies fail to deal with customer satisfaction, their future will be uncertain. Similarly, Hamzah and Shamsudin (2020) reported that customer satisfaction is essential for organizations to ensure market share and survival.

Furthermore, customer satisfaction act as a backbone of the company’s profit by increasing sales and bringing new customers into the business. Besides, an organization needs to understand their customer needs and expectations and bring as many improvements in products and services as possible to maintain its customers. In line with Müller (1991), top companies stated that we work for customers as customer satisfaction is our distinct objective. The way business is changing; our distinct objective is customer satisfaction. Moreover, they believe that customer satisfaction is the only significant variable that acts as a profit stimulant. Razak et al., 2016 in 2016 investigated the factors of customer satisfaction inconvenience products, i.e., toothpaste. The researcher collected the data from 110 respondents of Bekasi, Indonesia. The research found that customer satisfaction is enhanced when the product is reliable and standardized in quality at an affordable price. Therefore, the customer is interested in buying the product and re-purchases it repeatedly. When the customer receives more value from what they paid along with quality services, the customer feels satisfied with the brand or company (Muhammad et al., 2016). Researchers researched Pakistan telecommunication and selected the students of 99 university students who are using Mobilink mobile services. The purpose was to understand the significance of customer satisfaction in the market of Pakistan telecommunication.

The study further realized that customer satisfaction is an important contributing factor to the success of an organization as customers advertise their experience and fetch customers to the business. On the contrary, Al-Msallam (2015) investigated factors and the importance of customer satisfaction in the banking sector of Syria. The outcomes of the investigation enlighten that when the product or service is in accord with customer expectations and a fair price is charged against that product or service only. An organization earns increased sales and profit from customer satisfaction and heads the organization toward success. On behalf of the abovementioned literature following hypothesis is generated:

***H3***: *E-business success during the COVID-19 crisis depends on a company*’*s commitment to client happiness*.

**FIGURE 1:**
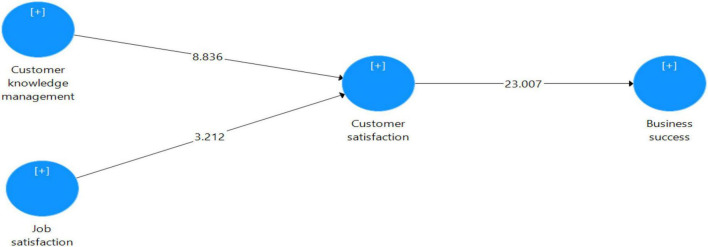
Exhibits the bootstrapping model. Source, Author’s constructed.

At the center of the overhead literature and outlined hypothesis, the study’s conceptual framework is formed, as shown in Figure 1.

## Methodology

### Research Design

This research uses Structural Equation Modeling (SEM) to examine the relationship between CKM, Job satisfaction, Customer satisfaction, and E-commerce success during a pandemic outbreak. SEM is the best statistical approach to evaluate the interrelated connections between multiple research variables in an advanced model. The primary data is used, and a Quantitative study is conducted through a self-administered survey questionnaire comprising demographic and multiple items for each of the four variables with taken 240 sample size of the research. The researchers contacted successful e-commerce players in Karachi and Lahore with an invitation to take part in the primary data analysis. In the study, successful e-commerce players are asked various questions to express their company’s success during COVID-19.

A Likert 5-point scale is used for the survey questionnaire, ranging from 1 as strongly agree to 5 as strongly disagree. The empirical models also take in job-related characteristics, i.e., education, experience in the current company, and designation. Age and gender are known to determine the employee’s commitment. SEM is the best statistical approach to evaluate the interrelated connections between multiple research variables in an advanced model.

### Statistical Techniques and Tools

Through SPSS, descriptive statistics have been executed from the primary data source, while reliability and validity of the instruments are measured by Cronbach alpha. The top fills the questionnaires, middle managers, and supervisors from their marketing, sales, production, human resource, and CKM departments.

The empirical models also take in job-related characteristics, i.e., education, experience in the current company, and designation. Age and gender are known to determine the employee’s commitment. This research employs SEM technique by using Partial Least Squares Structural Equation Modeling (PLS-SEM) approach kept up by Smart-PLS software and evolved a model that signifies the four main variables of the study, i.e., CKM, Job satisfaction and Customer satisfaction and E-commerce success to examine the relationship between first three variables and E-commerce success in the proceeding pandemic outbreak settings. According to Raines-Eudy (2000) and Bag (2015), SEM is the best statistical approach to evaluate the interrelated connections between multiple research variables in an advanced model. PLS path modeling is a distribution-free test practice that keeps down the aggregate of unexplained variation (Hair et al., 2017).

Furthermore, it facilitates the accountability for observational errors by using the weighted composites of indicator factors. Besides, a Quantitative study is conducted through a self-administered survey questionnaire comprising demographic and multiple items for each of the four variables of the research. Quantitative methodology is selected as it uses statistical data, which saves time and resources and is flexible to collect data from a large population (Daniel, 2016). Through SPSS, descriptive statistics have been executed, while the reliability and validity of the instruments are measured by Cronbach alpha. Hence, during quarantine quantitative research approach is an easy way to access participants.

### Sampling and Sample Size

In accord with the sampling techniques Taherdoost (2016) recommended, researchers collected data sample size is 240 from the fashion brands of Pakistan running successful online outlet stores operating in Karachi and Lahore. The reason behind targeting the fashion industry is that according to ecommerceDB (2020) statistics, 70% of Pakistan’s e-commerce revenue is generated through the fashion industry. The researchers contacted the successful e-commerce players in the fashion industry of Karachi and Lahore with an invitation to take part in the analysis. Hence, the researcher selected only those e-commerce players who maintained their success during the COVID-19 outbreak. After their approval, a survey questionnaire is sent to them. The top fills the questionnaires, middle managers, and supervisors of the ten successful e-commerce businesses from their marketing, sales, production, human resource, and CKM departments to recognize their business strategies regarding CKM, Job satisfaction, Customer satisfaction, and E-commerce success during COVID-19 crises. Almost 250 questionnaires were distributed but only 240 questionnaires were received.

### Measurements

#### Dependent Variable

Research is integrated to explain how CKM and job satisfaction act as an infrastructure of absorptive capacity which enables traditional small medium enterprises to successfully respond to the changing economic environment (like the COVID-19 crisis shifted the seller’s behavior) by satisfying varying customer demands and needs. To this end, the current study employs the dependent variable “**e-commerce successes**”. The successful e-commerce players are asked various questions to express their company’s success during COVID-19. To measure e-commerce success, five questions were extracted from the scale of Wach (2010). A Likert 5-point scale is used for the survey questionnaire, ranging from 1 as strongly agree to 5 as strongly disagree. Scale reliability is measured through Cronbach alpha, i.e., 0.822, indicating high reliability above the generally acceptable criterion of 0.70.

### Independent Variable

#### Customer Knowledge Management

Five items were averaged and used as a 5-point Likert scale (1 = “Strongly agree” to 5 = “Strongly disagree”) to estimate CKM as the infrastructure necessary for absorptive capacity. Questions were extracted and adjusted to better fulfill the purpose of the data collection from the scales of Taherparvar et al. (2014). Scale reliability was measured by Cronbach’s alpha, indicating the high reliability, i.e., 0.936.

#### Job Satisfaction

Based on Aziz and Lodhi (2015), Nanjundeswaraswamy (2019), research employed five items to measure job satisfaction and infrastructure necessary for absorptive capacity as a 5-point Likert scale (1 = “Strongly agree” to 5 = “Strongly disagree”). Scale reliability was measured by Cronbach’s alpha, indicating the high reliability, i.e., 0.858.

#### Customer Satisfaction

Five items were used as a 5-point Likert scale (1 = “Strongly agree” to 5 = “Strongly disagree”) drawn from the scales of Rahimić and Uštović (2012) with scale reliability of Cronbach’s alpha, i.e., 0.809.

### Control Variables

To isolate the outcomes of current research related variables, the empirical models also take in a set of job-related characteristics, i.e., education, experience in the current company and designation; demographic aspects, i.e., age, gender that are known to determine the employee’s commitment (Yaghoubi et al., 2011; Siva Kumar, 2014; Khuwaja et al., 2020).

## Data Analysis

Table 1 demonstrates the descriptive statistics of the respondent’s demographic features, performed using SPSS.20 software. Out of 240 respondents, 135 (56.3%) were male while 105 were women (43.8%); 87 (36.3%) were 23–28 years old, 114 (47.5%) were 29–34 years old, and 39 (16.3%) were 35–40 years old. Furthermore, 75 (31.3%) respondents held a bachelor’s degree, 131 (54.6) were with master’s degree, and 34 (14.2%) were diploma holders. In terms of experience in the current organization, 200 (83.3%) respondents were under 1–5 years, and 40 (16.7%) were in 6–10 years of experience. Regarding departments in the current organization, 48 (20%) were from sales, 49 (20.4%) were from marketing, 50 (20.8%) were from production, 48 (20%) were from CKM, and 45 (18.8%) were from human resource departments. Furthermore, the designation of the respondents in the current organization was 37 (15.4%) were top managers, 156 (65%) were middle managers, and 47 (19.6%) were supervisors.

**TABLE 1:** Descriptive statistics summary of the sample (*N*) = 240.

Variables	Mean	Std. deviation
Gender	1.4375	0.49712
Age	1.8000	0.69787
Qualification	1.8292	0.65327
Designation	2.0417	0.59137
Department	2.9708	1.40053
Experience	1.1667	0.37346
Quality of the current product/service	1.0417	0.20024
Customer’s background, purchasing capacity	1.0500	0.21840
Customers about the benefits of the current product and service	1.0958	0.29498
Better decisions and solve the customer’s problems more efficiently	1.0583	0.23486
Gaining customer’s ideas	1.0458	0.20956
Employee job satisfaction is	1.0708	0.25708
Performance-based-promotion gives the employees a	1.0708	0.25708
Customer’s inquiries	1.0958	0.29498
Training and career development opportunities	1.0625	0.24257
Secured job policies	1.0708	0.25708
Products/services are created exclusively	1.0625	0.24257
Customers the benefits	1.0792	0.27056
Offer quality products/services	1.0833	0.27696
Products/services our customers need	1.0708	0.25708
Complaints	1.2083	0.40697
Return on sales and sales growth	1.0542	0.22682
Profit growth	1.0417	0.20024
Increase in market share	1.0625	0.24257
The industry sector represents our business success	1.2417	0.42899
Business performance and growth	1.1667	0.37346

Additionally, the potential relationship between variables is also analyzed using the SPSS.20, which demonstrated adequate values with accuracy between 1.897 and 1.450 for kurtosis while –1.152 and –1.586 for skewness, which is an acceptable range (Matore and Khairani, 2020). The Spearmen’s correlation coefficient test presented adequate values showing a significant association between two variables, i.e., 0.957–0.838. Additionally, Kaiser-Meyer-Olkin (KMO) of sample adequacy and Bartlett’s Test of sphericity are executed to weigh the factorability. KMO value is 0.860 greater than 0.7, and Bartlett’s test shows the approximate chi-square value is 967.593, degree of freedom is six, and significance level is 0.000, less than 0.05 demonstrating that the scale is suitable for factor analysis (UlHadia et al., 2016). Moreover, in accord with Hair et al. (2017), for the credibility of the reflective measurement model, it is essential to examine the internal consistency (Cronbach’s alpha, Composite Reliability), convergent validity, and Discriminant Validity. Convergent validity is executed by Average variance extracted (AVE) as convergent validity defines the extent of variance captured by latent variables (which are not directly observed) from their related manifest variables (directly observable and required by the latent variable to observe) due to measurement errors, however, discriminant validity confirms that the manifest variable in any construct is related to the designated latent variable (Memon and Abdul Rahman, 2014).

Table 2 shows the results, and the researcher finds out that the four reflective measurement models bring off the admissible assessment criteria. Out of the 20 measurement items, three indicators show a moderate relationship, i.e., equal and above 0.4, while 17 indicators show a strong relationship, i.e., above 0.70, and exhibit an ample level of reliability (Ratner, 2009; Schober et al., 2018). More specifically, all AVE values are above 0.50, henceforth ideally supporting the convergence ability of the latent variables. The composite reliability of all the four measurement models is 0.868 and greater, above the minimum acceptable level of 0.70 (Saeed and Kassim, 2017; Gu et al., 2019). Into the bargain, Cronbach’s alpha values range between 0.809 and 0.936; this is quite above the acceptable range (Taber, 2018). These results put forward that the construct measures are pretty reliable with significant internal consistency.

**TABLE 2:** Reliability analysis results.

Variables	Items	Convergent validity	Internal consistency reliability
		Loadings AVE > 0.50	Cronbach’s CR alpha > 0.70 > 0.70
Customer knowledge management (CKM)	CKM1	0.987 0.803	0.936 0.949
	CKM2	0.908	
	CKM3	0.711	
	CKM4	0.858	
	CKM5	0.945	
Job satisfaction (JS)	JS1	0.774 0.638	0.858 0.898
	JS2	0.768	
	JS3	0.712	
	JS4	0.818	
	JS5	0.776	
Customer satisfaction (CS)	CS1	0.817 0.574	0.809 0.868
	CS2	0.737	
	CS3	0.703	
	CS4	0.779	
	CS4	0.451	
Business success (BS)	BS1	0.869 0.607	0.822 0.878
	BS2	0.987	
	BS3	0.819	
	BS4	0.455	
	BS5	0.487	

*CR is Composite reliability, while reliability, validity, and spearmen’s correlation coefficient tests are performed by using SmartPLS.*

After that, the researcher assesses the discriminant validity using the new criteria in variance-based SEM, i.e., HTMT_inference_ (Henseler et al., 2014), while HTMT is Heterotrait-Monotrait. Subsequently, it requires performing bootstrapping confidence intervals with 5,000 samples to test the HTMT_inference_ statistical significance of discriminant validity (Sarstedt et al., 2017). Table 3 shows discriminant validity results following the threshold of HTMT_inference_, demonstrating that discriminant validity is well established. Table 4 presents the bootstrapping results; it shows the statistical significance of path coefficient, HTMT, and *R*^2^ values.

**TABLE 3:** Discriminant validity results (HTMT_inference_).

	Business success	Customer knowledge management	Customer satisfaction	Job satisfaction
Business success customer knowledge management Customer satisfaction Job satisfaction	0.779			0.799
	0.935	0.896		
	0.865	0.893	0.758	
	0.893	0.914	0.857	

*SmartPLS perform a discriminant validity test.*

**TABLE 4:** General model resolution using the PLS algorithm and Bootstrapping.

Hypothesis	General framework	Effect	Path coefficient	Sample mean	Std. dev	*t*-value	*p*-value	*F* ^2^
H1	CKM → CS	Direct	0.664	0.665	0.075	8.836	0.000	0.374
	CKM → BS	Indirect	0.574	0.575	0.074	7.776	0.000	
H2	JS → CS	Direct	0.25	0.248	0.078	3.212	0.000	0.054
	JS → BS	Indirect	0.217	0.215	0.069	3.138	0.002	
H3	CS → BS	Direct	0.865	0.864	0.038	23.007	0.001	2.962

*H1, H2, and H3 all are supported.*

**FIGURE 2:**
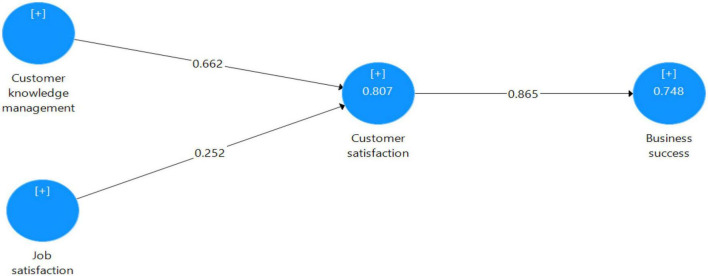
Results of the coefficient of variance *R*^2^. Source, Author’s constructed.

Bootstrapping is a non-parametric technique that computes *t*-value by generating a suggested number of samples. The acceptable *t*-values for a two-tailed test are 1.65 (significance level = 10%); 1.96 (significance level = 5%), and 2.58 (significance level = 1%). Bootstrapping test results in Table 4 reveal that all the paths that achieved *t*-value are greater than the cut-off point for a significance level of 1%. This implies that H3 has a strong effect with high value e. 0.865 (*t*-value = 23.007; *p* = 0.001) which is followed by H1 and H2 with value 0.664 (*t*-value = 8.836; *p* = 0.000) and 0.25 (*t*-value = 3.212; *p* = 0.000), respectively (Figure 2). Hence, the results in Table 4 also demonstrate the indirect effect of H1 and H2; both hypotheses are supported with *t*-value0.574 (*t*-value = 7.776; *p* = 0.000) and value 0.217 (*t*-value = 3.138; *p* = 0.002). Table 4 also shows the effect size (*f*^2^), which is important to understand (Khalilzadeh and Tasci, 2017). According to Hair et al. (2017), the coefficient of determination R2 and the effect size determine the capacity and strength of relationships between construct variables (f2). The researcher must additionally indicate the magnitude of the impact between variables and the significant level (Chin et al., 1996). The coefficient of determination *R*^2^ and adjusted *R*^2^ value for the Business success latent variable are 0.748 and 0.747, respectively, while *R*^2^ and adjusted *R*^2^ value for the Customer satisfaction latent variable are 0.807 and 0.806, correspondingly. It shows that Customer satisfaction moderately explains 74.7% (strong effect) of the variance in Business success. The two latent independent variables, i.e., CKM and Job satisfaction, moderately explain the 80.6% (strong effect) of the variance in customer satisfaction. Hence, Table 4 presents the results of (*f*^2^), which predictsH3, i.e., CS → BS, is supported with a high effect size of 2.962; *p* = 0.001, whereas H1, i.e., CKM → CS, is also supported with a high effect size of 0.374; *p* = 0.000 while H2, i.e., JS → CS is supported with a weak effect size of 0.054, but significance level is high, i.e., *p* = 0.000 (Henseler et al., 2009).

## Discussion and Practical Implication

COVID-19 is a pandemic that began in Wuhan, China, but continues to impact the emotional, social, personal, and professional lives of individuals worldwide despite its onset. Customers, businesses, and supply chains in several industries have been disrupted by using these sound effects. The 2020 pandemic’s global recession was either precipitated or prolonged by worldwide policies to control the global health issues posed by the coronavirus (Ozili and Arun, 2020). Happy customers act as one-to-one advertisers and help the company earn more money. A CKM system that is more extensive during the COVID-19 crisis results in a higher level of customer satisfaction.

An engaged workforce anticipates and responds swiftly to changing consumer demands and expectations, making them more devoted to their companies’ success. To achieve and maintain long-term success in business, entrepreneurs must please their clients. When a product is dependable, standardized, and reasonably priced, customers are more likely to be satisfied. Surveys are being conducted in Karachi and Lahore to gather information on CKM, job satisfaction, customer happiness, and the success of e-commerce businesses during the COVID-19 crisis. The four reflective measuring models meet the permissible evaluation requirements. The composite reliability of all the four measurement models is 0.868 and greater, above the minimum acceptable level of 0.70 (Saeed and Kassim, 2017; Gu et al., 2019). The empirical models also take in job-related characteristics, i.e., education, experience in the current company, and designation. Age and gender are known to determine the employee’s commitment. As a result of social media and its online applications, students can continue their education, households can be entertained, and quarantine stress can be reduced. The study’s findings strongly support the concept. TSMEs must flip the coin by successfully responding to the changing business climate and satisfying clients to enjoy the game instead of leaving the game. Business success and failure are two sides of the same coin. Customer happiness accounted for 74.8% of the variance in e-commerce performance. In contrast, the CKM and job satisfaction accounted for 80.7% of the variance in customer satisfaction, leaving 25.2 and 19.3% of the variance unaccounted for. Small-to-medium-sized businesses in Pakistan saw significant economic activity shifts during the COVID-19 financial crisis, which was the focus of this study.

Job satisfaction among employees is an essential resource of realized capacity to efficiently enjoy the objectives of absorptive capacity because external knowledge is needed to exploit organizations economically. 31% of entrepreneurs in Pakistan completely shut down their business activities, 43% of entrepreneurs lay off their employees, and 73.9% of Pakistani customers are unsatisfied with their online shopping. This study also provides some insight essential for traditional small-medium entrepreneurs, i.e., they need to manage their customer knowledge and employee satisfaction to cope well with the changing consumer-producer behavior.

## Conclusion

Global initiatives to tackle the global health challenges posed by the coronavirus either sparked or extended the global recession of the 2020 pandemic (Shehzad et al., 2021). During the COVID-19 crisis, a more thorough CKM system leads to a greater degree of customer satisfaction. The engaged workforce is more committed to the success of the company because it predicts and reacts quickly to changing consumer needs and expectations. Customers are more likely to be happy when a product is trustworthy, standardized, and competitively priced. During the COVID-19 crisis, when surveys are being done in Karachi and Lahore to collect data on CKM, job satisfaction, customer contentment, and the performance of e-commerce enterprises. The four reflecting measurement models all fulfill the assessment criteria which have composite dependability of 0.868 or higher. Job-related criteria, such as education, experience in the present business, and designation, are also factored into the empirical models. Lockdowns sparked by the outbreak in several nations have radically changed the globe in just a few weeks. For new customers, online shopping has become a painful experience, as they are frequently unsatisfied with the quality of their items and the astronomically high costs they pay. Traditional business owners must figure out how to deal with internet commerce in the future and what precautions to take to keep their businesses functioning well instead of fully shutting them down. A CKM system (COVID-19) is a crucial realized capability of a company with absorptive capacity. They may successfully use what they have learned and learned to achieve various degrees of client satisfaction.

## Data Availability Statement

The raw data supporting the conclusions of this article will be made available by the authors, without undue reservation.

## Author Contributions

AK and SA: conceptualization, introduction, methodology, interpreted results, writing–original draft preparation, and review and editing. ZC and SK: visualization, validation, conclusion, and writing–original draft preparation. FK and AN: conceptualization, formal analysis, project administration, supervision, and finalizes manuscript and review and editing. AK and LC: literature review, formal analysis, and review and editing.

## Conflict of Interest

The authors declare that the research was conducted in the absence of any commercial or financial relationships that could be construed as a potential conflict of interest.

## Publisher’s Note

All claims expressed in this article are solely those of the authors and do not necessarily represent those of their affiliated organizations, or those of the publisher, the editors and the reviewers. Any product that may be evaluated in this article, or claim that may be made by its manufacturer, is not guaranteed or endorsed by the publisher.
